# Early Atherosclerosis in Familial Hypercholesterolemia Patients: Significance of Vascular Markers for Risk Stratification

**DOI:** 10.3390/jcdd11030091

**Published:** 2024-03-13

**Authors:** Urte Aliosaitiene, Zaneta Petrulioniene, Egidija Rinkuniene, Antanas Mainelis, Jurate Barysiene, Urte Smailyte, Vaida Sileikiene, Aleksandras Laucevicius

**Affiliations:** 1 Faculty of Medicine, Vilnius University, LT-01513 Vilnius, Lithuania; zaneta.petrulioniene@santa.lt (Z.P.); egidija.rinkuniene@santa.lt (E.R.); jurate.barysiene@santa.lt (J.B.); urtesmailyte@gmail.com (U.S.); sileikiene.vaida@gmail.com (V.S.); cardio@santa.lt (A.L.); 2 Clinic of Cardiac and Vascular Diseases, Vilnius University Hospital Santaros Klinikos, LT-08661 Vilnius, Lithuania; 3 Faculty of Mathematics and Informatics, Vilnius University, LT-01513 Vilnius, Lithuania; amgpastas@gmail.com

**Keywords:** familial hypercholesterolemia, carotid intima–media thickness, pulse wave velocity, flow-mediated dilation, ankle–brachial index, cardio-vascular index

## Abstract

BACKGROUND: Familial hypercholesterolemia (FH) is a genetic disorder that manifests as impaired low-density lipoprotein cholesterol (LDL-C) metabolism, resulting in lifelong exposure to high cholesterol levels and increased risk of cardiovascular disease (CVD). There is heterogeneity in cardiovascular risk for FH patients, so risk stratification is of utmost importance. The aim of this study was to evaluate the impact of increases in LDL-C and the impact of other CVD risk factors on vascular markers in the FH patient population. METHODS: A total of 428 patients were included in this study and divided into two groups according to age: ≤40 years in the first group and ≥41 years in the second group. Vascular markers of atherosclerosis included the common carotid artery (CCA) intima–media thickness (IMT), pulse wave velocity (PWV), flow-mediated dilation (FMD), ankle–brachial index (ABI), and cardio-vascular index (CAVI). The influence of traditional CVD risk factors on atherosclerotic changes in vascular markers was analyzed. RESULTS: A statistically significant difference in IMT was detected between the same sex and different age groups (*p* < 0.001), whereas no significant difference was detected between the sexes within each age group. In the ≤40-year-old group, the mean IMT among males was 612.5 μm (±88.2) and that among females was 580.6 μm (±77.7) (*p* > 0.05); in the ≥41-year-old group, the mean IMT was 697.4 μm (±138.4) for males and 700.3 μm (±114.4) for females (*p* > 0.05). Higher LDL-C was associated with greater IMT (*r* = 0.405; *p* = 0.009) in the younger age group (≤40 years); however, in the older age group (≥41 years), this correlation was not evident (*r* = −0.07; *p* = 0.596). Carotid plaque formation was more common among males (OR = 2.2; 95% CI: 1.2–4.0) and hypertensive patients (OR = 2.7; 95% CI: 1.6–4.7). Age was a mildly significant risk factor for increased ABI (*β* = 0.13, *p* < 0.05). FMD was found to be impaired for all patients, and no risk factors were shown to have further influence. Age was a significant risk factor for increased arterial stiffness, as measured by both the CAVI and PWV. Conclusions: Although vascular markers of atherosclerosis may provide a unique and valuable way to evaluate cardiovascular risk, the results of this study show that only increased IM thickness could be beneficial for risk stratification in young FH patients, whereas other vascular markers of atherosclerosis would be excessive, as they do not provide merit in risk evaluation in this population.

## 1. Introduction

Familial hypercholesterolemia (FH) is an autosomal dominant disease that affects the metabolism of cholesterol and, therefore, causes a lifelong increase in low-density lipoprotein levels in patients. Recent studies have shown that despite the historical belief that the prevalence of FH is 1:500, the real prevalence is much closer to 1:300 [1]. Moreover, FH is universally underdiagnosed, as less than 1% of cases of FH are detected in most countries [2]. Due to such constant exposure to high blood levels of low-density lipoprotein cholesterol (LDL-C), FH patients have an increased risk of early atherosclerosis and premature major cardiovascular events. If left untreated, men suffering from FH have a 50% risk of fatal or nonfatal manifestation of coronary heart disease (CHD) by the age of 50, whereas untreated women have a 30% risk of CHD by the age of 60 [3]. Furthermore, cardiovascular disease is 3.5 times more prevalent in the FH population than in the general population [4]. Even more concerning, studies have shown that atherosclerotic vascular changes may be present in FH patients even in childhood [5]. Therefore, cardiovascular risk stratification should be one of the most important steps in the evaluation of FH patients, and vascular markers may provide a unique opportunity for the noninvasive evaluation of atherosclerosis in each patient. Early atherosclerosis can be assessed by surrogate vascular markers such as carotid intima–media thickness (IMT), pulse wave velocity (PWV), flow-mediated dilation (FMD), and the ankle–brachial index (ABI) or cardio-vascular index (CAVI). Nonetheless, comprehensive studies evaluating vascular markers of early atherosclerosis in FH patients are rare, and their routine use for patients with FH is still controversial. Several risk factors are recognized to facilitate the development of atherosclerosis: increasing age, smoking, male sex, hypertension, diabetes mellitus (DM), unhealthy diet, lack of physical activity, and dyslipidemia [6]. This knowledge puts patients with FH in a unique position since the concurrent effects of both constant exposure to high LDL-C and lifestyle factors affect this group. Studies on vascular markers of early atherosclerosis are needed to better understand the development and possible prevention of early atherosclerosis in patients with FH. Therefore, in this study, we aimed to determine the associations of CVD risk factors, especially increases in LDL-C, with vascular markers of early atherosclerosis in patients with clinically diagnosed FH.

## 2. Materials and Methods

### 2.1. Study Design

**Figure 1:**
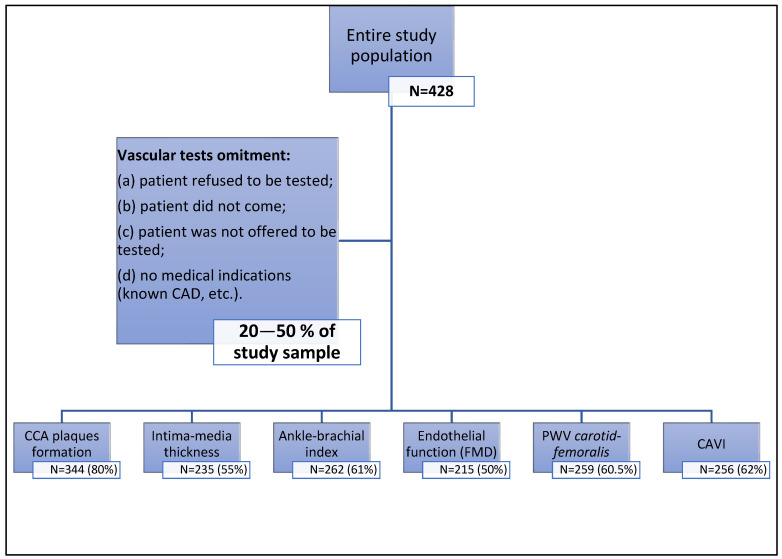
Selection of the patients for vascular markers testing. CCA—common carotid artery; FMD—flow-mediated dilation; PWV—pulse wave velocity; CAVI—cardio-ankle vascular index; CAD—coronary artery disease.

The Lithuanian National FH Screening Program started in 2016 and is mainly based on the Lithuanian High Cardiovascular Risk Primary Prevention Program (LitHir). The study was approved by the Vilnius (Lithuania) regional bioethics committee (permit number 158200-18/5-1010-538, issued 18 May 2018). According to the program, patients with FH are thoroughly examined, and cascade screening of first-degree relatives is initiated once an index case is detected. Data gathered from patients who signed a written informed consent form were entered into the long-term observation programme of FH in Lithuanian patients, as well as into the European Atherosclerosis Society (EAS) Familial Hypercholesterolemia Studies Collaboration (FHSC) global registry. Detailed examination of a potential FH patient includes gathering accurate personal and familial anamnestic data, extensive laboratory and instrumental testing, and consultations from other specialists: geneticists, radiologists, and ophthalmologists. The clinical diagnosis of FH was established according to the Dutch Lipid Clinic Network (DLCN) criteria. Molecular diagnosis is made after genetic testing, which is performed from a dried blood spot. After being enzymatically fragmented, genomic deoxyribonucleic acid (DNA) regions of interest are enriched using DNA capture probes. Next-generation sequencing was performed, as the final indexed libraries were sequenced on an Illumina platform. Patients were also tested for vascular markers of early atherosclerosis, including (1) arterial stiffness, which was assessed by carotid-radial and carotid-femoral artery pulse wave velocity; (2) endothelial function, which was assessed by flow-mediated dilatation; and (3) ultrasound, which was used to measure carotid artery intima–media thickness and assess atherosclerotic plaques in the carotid artery. Each patient included in the Lithuanian long-term observation programme of FH, as well as in the EAS FHSC global registry, was consulted by cardiologists (lipidology specialists). Not all consulted patients involved in the study have been tested for vascular markers for the following reasons: (a) All tests take a long time during the visit, which is why only the detection of carotid plaques was performed for most patients, as this test is mandatory for primary prevention patients, whereas other vascular tests are only optional. Therefore, some patients refused to participate in other tests, usually due to lack of time. (b) Some of the vascular tests were offered for the next day, but some patients did not come to perform optional tests. (c) In patients who had already been diagnosed with cardiovascular disease (ischemic heart disease, etc.), vascular tests were usually not performed. (d) A small part of the optional tests were not performed because the consulting physician did not offer them to their patients (Figure 1).

During the analysis, patients were divided into groups based on age. The first group comprised patients who were 40 years old or younger at the time of FH diagnosis and those who were older than 40 years. This allocation was chosen because age is an independent risk factor for cardiovascular impairment despite the patients’ risk profile. 

### 2.2. Measurement Methods

IMT measurements were performed with the patient lying on their back, with the neck rotated to the opposite side of the examination. The far wall of the CCA, free of atherosclerotic plaque with clearly visualized intima–media interfaces, was assessed longitudinally, at least 5 mm below the distal end of the CCA. IMT of the common carotid artery was measured using B-mode high-resolution echo-tracking technology ((Art.Lab, Esaote Europe B.V., Maastricht, The Netherlands). Bilateral measurements were performed and the average value was further analyzed.

ABI and CAVI were both calculated using the Fukuda Vascular Screening system VaSera VS-1000 (Fukuda Denshi Co., Ltd., Tokyo, Japan). The pressures and waveforms of ankle and brachial arteries, together with ECG and phonocardiograph, were measured, and the CAVI values were automatically calculated by the VaSera VS-1000.

Arterial stiffness was assessed by applanation tonometry (SphygmoCor, AtCor Medical, v.8.0, Sydney, Australia) system with a high-fidelity micromanometer (Millar R, Millar Instruments, Houston, TX, USA), which is placed on the skin at the projection of radial, carotid and femoral arteries in order to obtain pulse pressure wave curves. Brachial blood pressure was recorded, and the distance between the surface markings of the sternal notch and the femoral artery was measured. An electrocardiogram is recorded simultaneously, which allows the system to compute the cfPWV.

The FMD was performed according to the method based on Celermajer et al. [7]. The brachial artery diameter was measured on a B-mode imaging ultrasound system (Logiq 700, General Electric, Chicago, IL 60661, United States). The computerized software program for image acquisition and analysis was used (CVI Acquisition v.1.0.0.3 and CVI Analysis v.1.0.0.1, 1998 Information Integrity Inc. Little Rock, AR, USA). The arterial diameter was measured between the intima/lumen interfaces of the anterior and posterior wall at the end-diastole (synchronized with the beginning of the R wave on the continuously recorded ECG). Scans were taken in the longitudinal plane 1–8 cm above the antecubital fossa.

## 3. Statistical Analysis

All the statistical analyses were performed using the R (v. 4.0.4) program package. Categorical variables are presented as the absolute amount and percentage. To test hypotheses for comparisons of quantitative variables between two groups, Student’s t tests or nonparametric Mann–Whitney U tests were used as appropriate. To test hypotheses for comparisons of quantitative variables between more than two groups, one-way analysis of variance (ANOVA) or the nonparametric Kruskal–Wallis test was used as appropriate. Normality was tested using the Shapiro–Wilk test. To test hypotheses for between-group comparisons of categorical variables, Pearson’s chi-square test or Fisher’s exact test was used as appropriate. To identify relationships between two quantitative variables, Pearson or Spearman correlation was used as appropriate. In order to test multiple variables relations, multivariate linear regression was used for quantitative variables with standardized beta coefficients. To test multiple variable relationships for the binary outcome (BMA), a multivariate logistic regression model was used. Only variables with statistically significant coefficients were kept in the final model, and Odds-Ratio (ORs) were estimated for them by taking exponential transformation of coefficients. A *p* value less than 0.05 was considered significant.

## 4. Results

A total of 428 patients were included in this study—228 females (53%) and 200 males (47%). The general characteristics of the study sample are presented in Table 1. A statistically significant difference in the median age of diagnosis between sexes was found: the median age of FH diagnosis for males was 43 years, whereas for females, it was 53 years (*p* < 0.005). Smoking was also significantly more common among male patients, where 40% of males were smokers, while only 25% of females were smokers (*p* < 0.005). No significant difference in median LDL-C or the occurrence of DM was found between the sexes. Thirty percent (*n* = 127) of patients were genetically tested for FH-causing mutations. Mutations related to FH were found in 38.6% (*n* = 49) of the genetically tested patients, while no mutations were detected in 61.4% (*n* = 78) of the subjects. The most common mutation was of the LDLR gene, found in 55% (*n* = 27) of patients, whereas the second most common mutation was of the ABOB gene and was found in 31% (*n* = 15) of all genetically proven cases. One patient had a mutation in both LDLR and APOB genes, and for six patients, data on the location of the mutation was not available.

### 4.1. Intima–Media Thickness (IMT) of the Common Carotid Arteries

**Figure 2:**
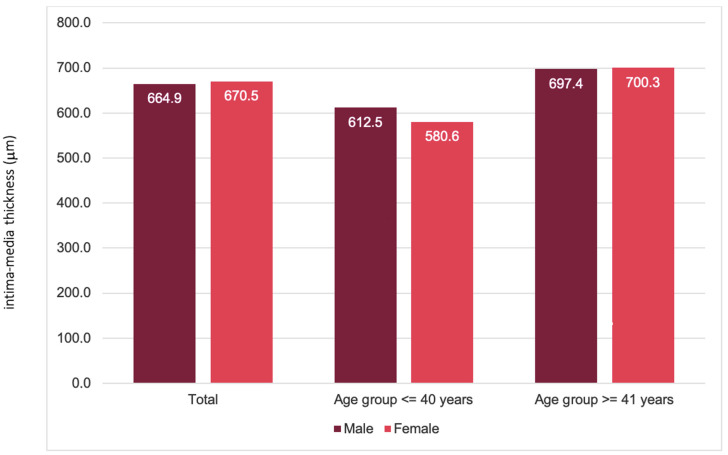
Differences in the mean intima–media thickness between sexes.

The first group (aged ≤ 40 years) consisted of 99 patients (54 males and 45 females), whereas the second group (aged ≥ 41 years) consisted of 236 patients (97 males and 139 females). The mean IMT in the entire study population was 668.99 μm (664.9 μm in males; 670.5 μm in females; *p* > 0.05). A statistically significant difference in IMT was detected between the same sex and different age groups (*p* < 0.001), whereas no significant difference was detected between males and females within each age group. In the ≤40-year-old group, the mean IMT was 598.0 μm (±84.7); among males, it was 612.5 (±88.2); and among females, it was 580.6 (±77.7) (*p* > 0.05). In the group ≥41 years, the mean IMT was 699.1 μm (±124.5); for males, it was 697.4 (±138.4); and for females, it was 700.3 (±114.4) (*p* > 0.05). The distributions of IMT between age groups and sexes are shown in Figure 2.

**Figure 3:**
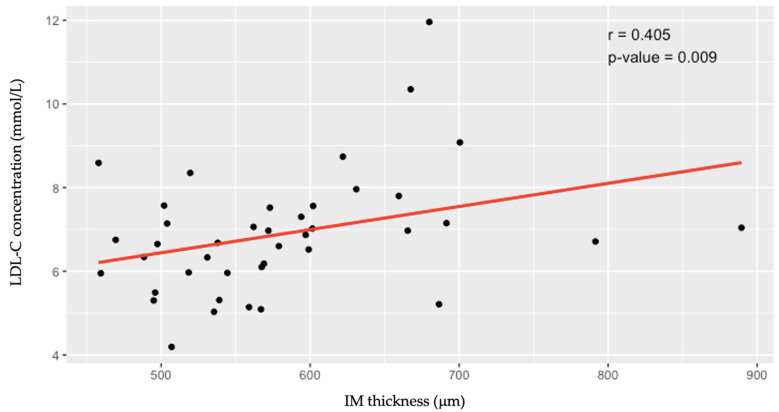
Associations between low-density lipoprotein cholesterol levels and the intima–media thickness of the common carotid artery in individuals aged ≤40 years. LDL-C—low-density lipoprotein cholesterol; IM—intima–media.

**Figure 4:**
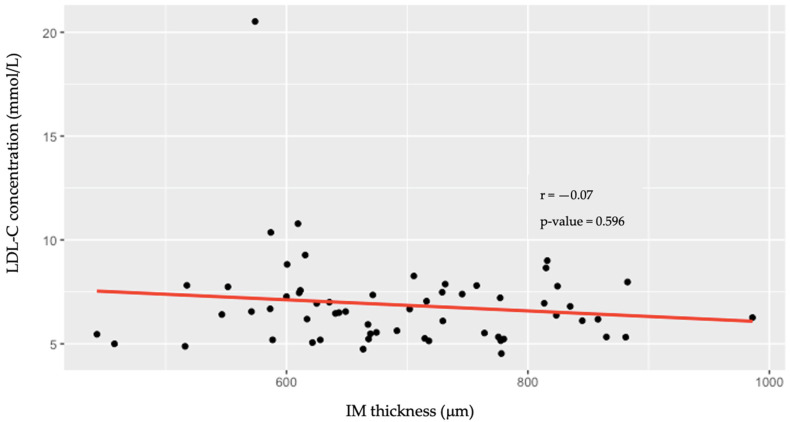
Associations between low-density lipoprotein cholesterol levels and the intima–media thickness of the common carotid artery in individuals aged ≥41 years. LDL-C—low-density lipoprotein cholesterol; IM—intima–media.

Higher LDL-C was associated with greater IMT (*r* = 0.405; *p* = 0.009) in the younger age group (≤40 years); however, in the older age group (≥41 years), there was no correlation (*r* = −0.07; *p* = 0.596) (Figure 3 and Figure 4). 

### 4.2. Common Carotid Artery (CCA) Plaque Formation

**Figure 5:**
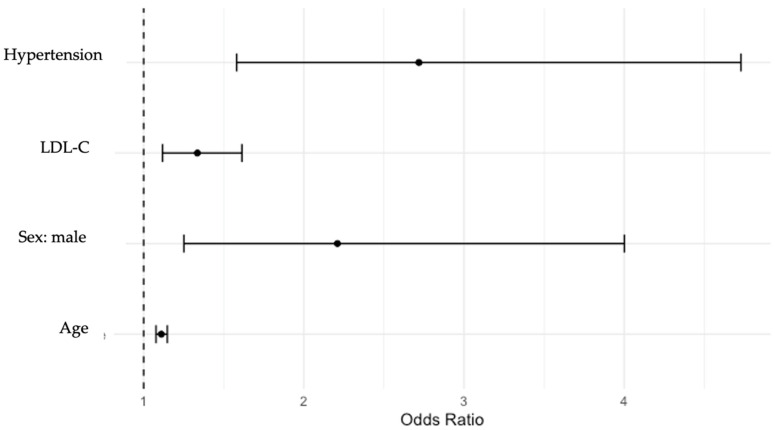
Associations between cardiovascular risk factors and common carotid artery plaque formation. LDL-C—low-density lipoprotein cholesterol.

A total of 157 males and 187 females were included in this subanalysis. CCA plaques were detected in 98 males (62%) and 118 females (63%), *p* > 0.05. The ORs indicated that within the analyzed population, carotid plaque formation was more common among males (OR = 2.2; 95% CI: 1.2–4.0) and hypertensive patients (OR = 2.7; 95% CI: 1.6–4.7). Age and an increase in LDL-C of 1 had a less significant impact (age: OR = 1.1; 95% CI: 1.07–1.15). LDL-C: OR = 1.3; 95% CI: 1.12–1.6). The associations between cardiovascular risk factors and CCA plaque formation are shown in Figure 5. Other risk factors, such as smoking status and DM status, did not significantly impact carotid plaque formation within the analyzed study sample.

### 4.3. The Value of the Ankle–Brachial Index (ABI) for Detecting Peripheral Artery Disease (PAD)

The subanalysis sample consisted of 116 males and 146 females. The mean ABI did not differ between the sexes, and the same value was recorded for both sexes (1.1 ± 0.1). Within the analyzed sample, increases in LDL-C did not significantly influence ABI changes, while age was the only mildly significant risk factor for PAD (*β* = 0.13, *p* < 0.05) in our model.

### 4.4. Endothelial Function Assessed by Flow-Mediated Dilation (FMD)

**Figure 6:**
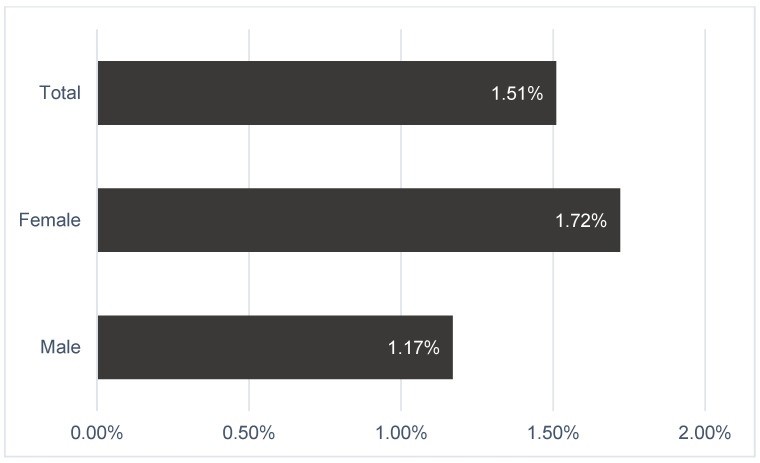
Distribution of flow-mediated dilation values within the study population.

The sample in this subanalysis consisted of 92 males and 123 females. FMD was impaired for all patients in the study sample and did not differ significantly between sexes: the median FMD in the entire population was 1.51 ± 1.65 %, whereas for females, it was 1.72% ± 1.8 and 1.17% ± 1.3 for males (*p* > 0.05). The distribution of FMD values within the study sample is shown in Figure 6. Among the analyzed FH patient population, neither LDL-C increase nor other risk factors, such as age, sex, DM, hypertension, obesity, or smoking, had a significant influence on further FMD changes.

### 4.5. Measurements of Arterial Stiffness

#### 4.5.1. Aortic Stiffness, Measured as Aortic Pulse Wave Velocity (PWV Carotid-Femoralis (*cf*PWV))

The subanalysis sample consisted of 116 males and 143 females. The median *cf*PWV did not differ between the sexes: the median *cf*PWV for males was 7.7% ± 1.4, whereas for females, it was 7.6% ± 1.6, *p* > 0.05. Within the analyzed sample, an increase in LDL-C did not significantly influence *cf*PWV, while only age and diabetes mellitus (DM) were significant risk factors for increased aortic arterial stiffness (accordingly, *β* = 0.43 and *β* = 0.18, *p* < 0.05).

#### 4.5.2. Arterial Stiffness, Assessed by the Cardio-Ankle Vascular Index (CAVI)

The study sample consisted of 112 males and 144 females. The mean CAVI did not significantly differ between sexes: the mean CAVI in males was 7.17 (±1.2), while in females, it was 7.5 (±1.0), *p* > 0.05. Within the analyzed sample, the increase in LDL-C had no significant influence on the CAVI. According to our model, only age was shown to be a significant risk factor for increased arterial stiffness, as measured by the CAVI (*β* = 0.47, *p* < 0.05).

## 5. Discussion

Patients with FH are at increased risk of cardiovascular events. However, the cardiovascular risk in the FH population is not uniform [8]. For instance, patients with an identified causative mutation are at greater cardiovascular risk than patients with polygenic FH [9]. Because clear guidelines on diagnosis and effective treatment options already exist, cardiovascular risk stratification is becoming one of the major concerns for FH patients. With more aggressive treatment options, such as pro-protein convertase subtilisin/kexin 9 (PCSK9) inhibitors available, risk stratification may provide the opportunity for a more personalized approach to FH treatment. Nevertheless, only a small proportion of patients with FH tend to achieve LDL-C goals [10]. Several personal and genetic characteristics are important for the evaluation of cardiovascular risk; however, instrumental tests that work as surrogate markers of atherosclerosis should not be overlooked. In this study, most clinically significant vascular markers of early atherosclerosis are reviewed in the FH population.

The intima–media thickness of the common carotid artery is a well-known indicator of cardiovascular risk because it reflects the thickening of the arterial wall caused by atherosclerosis. It has been proven that IMT correlates with the risk of both coronary syndrome and ischemic stroke [11,12]. Several risk factors are thought to affect IMT values: age, sex, smoking status, hypertension status and blood cholesterol levels [13,14,15]. Multiple studies have investigated IMT in children with FH and have shown that children with FH have a greater IMT than healthy controls, but the progression of IMT can be significantly slowed by statin therapy [16,17]. The results of this study showed that age is an independent risk factor for IM thickening, whereas higher LDL-C was an independent risk factor for higher, still within the normal range, IMT only in younger patients (aged ≤40 years). The finding that age is a risk factor for increased IMT is supported by the study by Ogura et al., as it was reported that both the mean and maximum IMT significantly increased with increasing years of follow-up [11]. It should be noted that despite differences in median IMT values between study groups, median IMT remained within the normal range with a tendency to increase in younger patients with higher LDL-C concentrations.

Another commonly used test for the assessment of cardiovascular risk is carotid ultrasound and the assessment of atherosclerotic plaque formation in the carotid artery. Carotid plaques are also strongly positively associated with cardiovascular risk [5,18]. In this study, carotid plaques were more common among males and hypertensive patients. Furthermore, higher LDL-C values were also associated with a greater risk of carotid plaque formation; however, this association was less significant than that of the previously mentioned risk factors. In contrast, Waluś-Miarka et al. found no significant differences in carotid plaque incidence among males and females or between carotid plaques and LDL-C levels among both sexes [18].

One of the earliest signs of atherosclerosis is vascular damage and dysfunction of the endothelium. Therefore, flow-mediated dilation, a novel method for evaluating endothelial dysfunction, may be particularly useful in cardiovascular risk stratification because it represents extremely early atherosclerotic damage. Common cardiovascular risk factors, including age, sex, smoking status, and hypertension status, are hypothesized to affect FMD [19]. The results of this study show that patients with FH have impaired endothelial function despite their CVD risk profile and LDL-C levels. In a study by Antonios P. Vlahos et al., it was shown that endothelial function in FH patients may be impaired as early as the age of 10 [20]. Other studies have also confirmed that patients with FH tend to have lower FMD values than healthy controls [21]. In their meta-analysis, Christian Heiss et al. showed that the average FMD in healthy individuals was 6.4%, and while FMD > 6.5 virtually excluded CAD, FMD values < 3.1% indicate very high cardiovascular risk [22]. Therefore, it may be hypothesized that the measurement of FMD in adult FH patients is robust and does not provide any useful insight into CVD risk stratification. On the other hand, other studies suggest that FMD does not correlate with clinical outcomes in patients without FH; thus, further comprehensive studies are required to better estimate the clinical importance of FMD [23].

Patients with FH may be at increased risk for not only CAD but also ischemic stroke and PAD [24,25,26]. However, patients are usually not screened for PAD once FH is suspected, contrary to screening for CAD. The prevalence of PAD has increased substantially over the decades, but it still often remains underdiagnosed [27]. The ABI is a commonly used test for diagnosing PAD. A study by Emanuelsson et al. showed that low ABIs may be associated with an increased risk of MI, even after adjusting for risk factors, which further promotes the use of the ABI as a risk assessment tool [28]. Multiple CVD risk factors are hypothesized to affect ABI [29]. In this study, we tested which risk factors had the greatest influence on the ABI. Our results showed that age had the greatest effect on the risk of PAD, whereas increasing LDL-C did not significantly increase the risk of PAD. However, it should be noted that most patients in our study were not obese or nonsmokers and did not have diabetes or hypertension, which, to the best of our knowledge, are considered to be the main risk factors for PAD. Thus, as per the results of this study, it could be proposed that FH patients without other risk factors should not be routinely tested for PAD. Nonetheless, some other studies have come to opposite conclusions; therefore, further research is undoubtedly needed [28].

Pulse wave velocity measures arterial stiffness and is a commonly used surrogate marker of atherosclerosis. Several studies have investigated arterial stiffness in the FH population, and no consensus on whether it should be a routine test for FH patients has been reached. However, whether carotid-femoral PWV is greater in patients with FH than in healthy controls is still controversial [30]. The risk factors that most commonly affect PWV are age, hypertension, diabetes mellitus, obesity, and smoking [31,32]. The results of this study showed that an increase in LDL-C in the FH population did not independently influence aortic arterial stiffness measured by *cf*PWV. Higher PWV values were observed in patients with diabetes mellitus and were also associated with greater age. PWV increase with age has also been proven in other studies [30]. Despite the fact that PWV is recognized as the gold-standard method for evaluating arterial stiffness, it is known to be strongly affected by blood pressure at the time of measurement. In contrast, the CAVI, a relatively neoteric method for the evaluation of systemic arterial stiffness, has been reported to be Independent of blood pressure and may, therefore, be a beneficial additional test representing the state of atherosclerosis in a patient [33]. The risk factors that are hypothesized to influence CAVI values are age, male sex, dyslipidemia, and DM [34]. However, very few studies investigating CAVI in the FH population exist. In this study, we investigated the impact of risk factors such as increased LDL-C levels, smoking status, and hypertension on the CAVI. Age was found to be the only risk factor for increased arterial stiffness, measured by the CAVI within the analyzed sample. The association between age and increased CAVI values is well supported by other studies [35]. Moreover, the lack of an association between other risk factors and CAVI in our study could also be explained by the relatively small sample size; therefore, further research is crucially needed.

## 6. Conclusions

Based on the results of this study, it is likely that only the measurement of IMT could be beneficial for FH patients, whereas other vascular markers may be excessive, as they provide little additional information. However, in our model, higher LDL-C values were associated with increased IMT in younger patients (≤40 years), and this finding may be useful for better risk stratification of young FH patients who usually have fewer risk factors and fall into a lower CVD risk category.

## 7. Study Strengths and Limitations

This study reviews most of the currently clinically relevant vascular markers of early atherosclerosis and their characteristics in an FH population. The analysis suggests that some of the vascular markers are significantly impaired (revealing early atherosclerosis in FH patients) and could help to stratify the cardiovascular risk of FH patients, especially those who tend to have a milder cardiovascular risk profile. However, there are several limitations to this study. First, it was a cross-sectional design, and the sample size was relatively small, with significant differences in patient count in the subanalyses, as performing all the vascular tests in patients was a technical and logistic challenge. Moreover, young adults, especially children with FH, are underrepresented in this study, as the median age of FH diagnosis is 47 years, which also means that for most patients, atherosclerosis may be advanced. Additionally, the model of this study does not take into account whether patients are receiving lipid-lowering therapy. Furthermore, this study did not include a control group; however, the reference values for the analyzed vascular markers have already been established. Therefore, the inclusion of healthy individuals in this study would have been a nonappropriate allocation of our resources. Lastly, due to the cross-sectional model of this study, only associations between risk factors and vascular marker values were investigated, which unfortunately does not allow for the evaluation of causality.

## Figures and Tables

**Table 1:** General characteristics of the study population.

	Total	≤40	≥41	*p* Value
N (%)	428	126 (29%)	292 (68%)	-
Median Age (years)	47	-	-	-
Median LDL-C (mmol/L ± SD)	6.37 ± 1.63	6.71 ± 1.7	6.23 ± 1.6	**0.034**
Definite FH (N)	83	46 (55.4%)	35 (42.2%)	-
Probable FH (N)	97	23 (23.7%)	72 (74.2%)	-
Possible FH (N)	224	49 (21.9%)	170 (75.9%)	-
Unlikely FH (N)	24	8 (33.3%)	15 (62.5%)	-
Hypertension (N)	221	36 (16.3%)	181 (81.9%)	**<0.001**
Diabetes mellitus (N)	44	4 (9.1%)	39 (88.6%)	**0.002**
Smoking (N)	128	35 (27.3%)	91 (71.1%)	0.686
FH mutation (N)	49	25 (51%)	23 (46.9%)	**0.006**

LDL-C—low-density lipoprotein cholesterol; FH—familial hypercholesterolemia; SD—standard deviation.

## Data Availability

The datasets used and analyzed during the current study are available from the corresponding author upon reasonable request.

## Abbreviations

ABI	ankle–brachial index
ANOVA	one-way analysis of variance
CAVI	cardio-vascular index
CCA	common carotid artery
*cf*PWV	*carotid femoralis* pulse wave velocity
CHD	coronary heart disease
CI	confidence interval
CVD	cardiovascular disease
DLCN	Dutch Lipid Clinic Network
DM	diabetes mellitus
DNA	deoxyribonucleic acid
EAS FHSC	European Atherosclerosis Society Familial Hypercholesterolemia Studies Collaboration
FH	Familial hypercholesterolemia
FMD	flow-mediated dilation
IMT	intima media thickness
LDL-C	low-density lipoprotein cholesterol
LitHir	Lithuanian High Cardiovascular Risk Primary Prevention Program
OR	odds ratio
PAD	peripheral artery disease
PCSK9	pro-protein convertase subtilisin/kexin 9
PWV	pulse wave velocity
SD	standard deviation
μm	micrometer
